# Socioeconomic deprivation and serious ocular trauma in Scotland: a national prospective study

**DOI:** 10.1136/bjophthalmol-2016-309875

**Published:** 2017-03-08

**Authors:** Liying Low, James Hodson, Daniel Morris, Parul Desai, Caroline MacEwen

**Affiliations:** 1 Academic Unit of Ophthalmology, University of Birmingham, Birmingham and Midland Eye Centre, Birmingham, UK; 2 University Hospitals Birmingham NHS Foundation Trust, Queen Elizabeth Hospital Birmingham, Birmingham, UK; 3 Cardiff Eye Unit, University Hospital of Wales, Cardiff, Wales, UK; 4 Moorfields Eye Hospital NHS Foundation Trust, London, UK; 5 Department of Ophthalmology, Ninewells Hospital and Medical School, University of Dundee, Dundee, UK

**Keywords:** Trauma, Public health

## Abstract

**Objective:**

To identify the population at risk of serious ocular trauma by exploring relationships with socioeconomic factors.

**Design:**

National, prospective, population-based, cross-sectional and follow-up study.

**Participants:**

Patients with serious ocular trauma requiring hospital admission in Scotland.

**Methods:**

*Case definition and ascertainment*—cases of serious ocular trauma necessitating admission to hospital under the care of a consultant ophthalmologist were identified using the British Ophthalmological Surveillance Unit reporting scheme. Using the postcode of residence, we assigned a Scottish Index of Multiple Deprivation (SIMD) score, SIMD quintile ( 0%–20% most deprived; 20%–40%, 40%–60%, 60%–80%, 80%–100% least deprived areas), geographical access score as well as the estimated travel time to the nearest general practitioner (GP) practice using either car or public transport for each patient. Population estimates were obtained from the General Register Office for Scotland.

**Main outcome measure:**

Serious ocular trauma requiring hospital admission.

**Results:**

A total of 104 patients (85.6% male) were reported as being admitted with ocular trauma with a median age of 32 years (IQR 24–54). There was a trend for increasing incidence of serious ocular injury with increasing socioeconomic deprivation (p=0.034). Patients from the most deprived areas (SIMD: 0%–20%) were twice as likely to sustain ocular injury compared with those from the least deprived (SIMD: 80%–100%) areas (relative risk: 2.19, 95% CI 1.02 to 4.81). There was no significant difference in the drive/public transport time to GP practices across the SIMD quintiles.

**Conclusions:**

Increasing socioeconomic deprivation was associated with a higher incidence of serious ocular injury. Targeted interventions are needed to address inequality in eye healthcare in deprived areas.

Ocular trauma is an important cause of preventable visual impairment globally.^1^ Sustained efforts to reduce the burden of this public health issue have led to changes in the aetiology of eye injuries. Following changes in legislation, the incidence of eye injuries occurring in the workplace due to road traffic accidents and during sporting activities has been reduced. As a result, causes of ocular trauma have moved from areas of corporate responsibility to those of personal responsibility.^2–4^

Health inequalities exist across the entire spectrum of the healthcare service, with increasing evidence to suggest that individuals living in deprived areas disproportionately experience the burden of trauma, paucity of access to eye and healthcare services, ill health, morbidity and mortality.^3–7^

To this end, we seek to identify the population at risk of serious ocular trauma and elucidate the relationship between socioeconomic deprivation and the incidence of serious ocular trauma in Scotland.

## Methods

### Data collection and definitions

We conducted a 1-year, population-based, prospective study of serious ocular trauma in Scotland.^2^ ^8^ We identified cases of serious ocular trauma through the British Ophthalmological Surveillance Unit (BOSU) active reporting scheme^9^ between 1 November 2008 and 31 October 2009. Every month, consultant ophthalmologists in Scotland reported newly diagnosed cases of serious ocular trauma—defined as ‘an injury or wound to the eye or adnexae caused by external force or violence, which requires admission to hospital for observation or treatment’.

The reporting ophthalmologist provided information about each patient on a structured form that included the basic demographic data, cause of injury, presenting features including intoxication and initial management of the injury. Intoxication was defined as any evidence of alcohol or recreational drug use at time of injury. We collected further data on outcome and secondary management of these cases from follow-up reporting forms sent out 12 months after the injury which are reported elsewhere.^2^ ^8^

### Population and healthcare provision in Scotland

We obtained the population estimates (mid-2009) from the General Register Office for Scotland. Population estimates in mid-2009 were 5 347 631 people. The framework for healthcare provision in Scotland is based on free universal care funded nationally by the Scottish government and provided by the National Health Service Scotland.

### Socioeconomic deprivation scores

We used the Scottish Index of Multiple Deprivation (SIMD) 2012 score as a measure of area-based deprivation. The SIMD score for each geographical datazone is a combination of 38 indicators of deprivation across seven broad domains: crime, geographical access, income, health, housing, education, skills and training. The larger the overall SIMD score, the more deprived the area.

We used residential postcodes to assign each patient a datazone, and their individual SIMD score. We grouped patients into quintiles: 0%–20% most deprived, 20%–40%, 40%–60%, 60%–80%, 80%–100% least deprived. This approach is consistent with that recommended by the Scottish Government Office of the Chief Statistician and Performance,^10^ and are used in other parts of the UK^11–15^ and elsewhere^16–18^ to categorise measures of deprivation.

### Geographical access and time to general practitioner

We used the residential postcode to identify the geographical access score, as a proxy indicator of access to healthcare services. The geographical access score captures the financial cost, time and inconvenience of accessing basic services, along with the driving and public transport times to the nearest general practitioners'(GPs)/family doctors' clinic.

We obtained ethics committee approval from the Newcastle and North Tyneside 1 Research Ethics Committee (Reference 08/H0906/70).

This research adhered to the tenets of the Declaration of Helsinki.

### Statistical analyses

We performed all statistical analyses using IBM SPSS V.22.0 (IBM. Armonk, New York, USA). Continuous variables were compared between groups using Mann-Whitney tests. Ordinal variables were analysed using Kendall's tau and reported as rates or relative risks (RR). Correlations were analysed using Spearman's rho. We excluded any missing data on a per-analysis basis and deemed p<0.05 to be indicative of statistical significance.

## Results

One hundred and four patients with serious ocular trauma were admitted to hospitals in Scotland during November 2008 to October 2009. The median age at time of injury was 32 years (IQR 24–54 years) and 89 (86%) patients were male.

### Completeness of case ascertainment

Consultant response to the BOSU reporting system was 77.1%. Good geographical coverage was achieved with all ophthalmic departments in Scotland participating in this study. Their collective catchment areas for presentation and referral of serious ocular trauma (as defined here) ranged from the far north in rural Scotland to the south borders.

### Socioeconomic deprivation

SIMD scores were identified for 98 (94%) patients for whom postcodes were available. There were no individuals identified as ‘of no fixed abode’. We were unable to assign SIMD scores to 6% of the cases due to incomplete postcode information from the questionnaires. Serious ocular injury necessitating admission to hospital was found to be significantly associated with socioeconomic deprivation (p=0.034). Patients from the 0%–20% most deprived areas were twice as likely to sustain serious ocular trauma compared with those from the 80%–100% least deprived areas (RR 2.19, 95% CI 1.02 to 4.81) (table 1).

**Table 1:** Ocular injuries by SIMD score

SIMD quintile	n	Ocular injuries	Rate (per 100 000)	RR* (95% CI)
0%–20% most deprived	999 853	22	2.20	2.19 (1.02 to 4.81)
20%–40%	1 040 302	22	2.11	2.10 (0.98 to 4.62)
40%–60%	1 082 867	23	2.12	2.11 (0.99 to 4.61)
60%–80%	1 130 008	20	1.77	1.76 (0.81 to 3.92)
80%–100% least deprived	1 094 601	11	1.00	–

Kendall's tau: p=0.034.

Based on n=98 with complete postcodes.

*Relative to the least deprived quintile.

RR, relative risk; SIMD, Scottish Index of Multiple Deprivation.

There was no statistically significant difference in the level of visual acuity at time of presentation or the final visual outcome within the SIMD quintiles, or the type of injury (table 2).

**Table 2:** Visual acuity and type of injury

		n	Overall SIMD score	p Value
Initial visual acuity	Better or equals to 6/12	25	20.0 (10.6–36.4)	0.697
	Worse than 6/12	66	18.3 (11.6–29.1)	
Final visual acuity	Better or equals to 6/12	49	17.7 (10.6–27.5)	0.473
	Worse than 6/12	36	19.1 (12.7–37.1)	
Type of injury	Blunt trauma	41	19.1 (12.7–29.9)	0.581
	Penetrating eye injury	44	19.5 (10.3–31.3)	
	Other	13	21.5 (14.5–37.1)	

Data reported as medians and quartiles, with p values from Mann-Whitney tests (visual acuity) and Kruskal-Wallis test (type of injury).

SIMD, Scottish Index of Multiple Deprivation.

### Gender, age and mechanism of injury

There was no statistically significant difference in the overall SIMD scores between the genders (male, median: 19.7 IQR: 10.9–30.9; female, 18.8, 13.1–36.4 (p=0.562) and age (Spearman's rho=−0.081, p=0.428).

### Intoxication

There was evidence of intoxication at the time of injury in 25 (26.9%) patients, all of whom were male. These patients were more likely to have resident postcodes in more deprived areas (p=0.004) and those having higher crime rates (p=0.010) (table 3).

**Table 3:** Intoxication at time of ocular injury

	Intoxicated		
	Yes (n=25)	No (n=63)	p Value
Overall SIMD score	25.0 (17.7–40.2)	15.2 (10.3–24.4)	0.004*
Geographical access score	20.6 (12.9–38.3)	19.4 (7.8–39.2)	0.630
Drive time (min)	4 (3–5)	4 (3–5)	0.640
Public transport time (min)	10 (8–12)	9 (7–16)	0.980
Crime (per 10 000)	493 (303–689)	249 (121–595)	0.010*

Data reported as medians and quartiles, with p values from Mann-Whitney tests.

Based on n=88 with complete postcodes and intoxication status.

*Significant at p<0.05.

SIMD, Scottish Index of Multiple Deprivation.

Both the mechanism and place of injury differed significantly with intoxication status (p<0.001), with intoxicated patients having higher rates of assault, and more likely to be injured in public areas (table 4).

**Table 4:** Mechanism and place of injury

	Intoxicated		
	Yes (%)	No (%)	p Value
Mechanism			
Assault	20 (87)	8 (12)	<0.001*
Fall	2 (9)	9 (13)	
Vehicle accident	1 (4)	1 (2)	
Other	0 (0)	32 (48)	
Machinery/tools	0 (0)	17 (25)	
Total	23 (100)	67 (100)	
Place of injury			
Public areas	20 (83)	8 (14)	<0.001*
Home	4 (17)	26 (44)	
Work/school	0 (0)	19 (32)	
Sports/leisure facility	0 (0)	6 (10)	
Total	24 (100)	59 (100)	

Data reported as n (column %), with p values from Fisher's exact tests.

*Significant at p<0.05.

### Time to presentation and geographical access

There was no statistically significant difference in the overall SIMD scores of patients presenting <24 hours and those presenting later (p=0.402). There was no significant association between SIMD score and the driving/public transport time to GP practices across the SIMD quintile, nor was there a significant difference in travel times between those who presented <24 hours from time of injury and those who presented later (table 5).

**Table 5:** Time to presentation

	Time to presentation		
	Less than 24 hours (n=83)	More than 24 hours (n=12)	p Value
Overall SIMD score	19.1 (11.6–29.9)	21.3 (11.7–62.2)	0.402
Geographical access score	19.0 (7.8–39.1)	20.5 (11.7–46.4)	0.533
Drive time (min)	3.7 (2.6–4.9)	4.3 (3.0–6.9)	0.269
Public transport time (min)	8.9 (6.7–14.3)	10.7 (7.9–17.8)	0.333

Data reported as medians and quartiles, with p values from Mann-Whitney tests.

Based on n=95 with complete postcodes and time to presentation status.

SIMD, Scottish Index of Multiple Deprivation.

## Discussion

In our study, there was a trend for increasing frequency of serious ocular trauma with increasing socioeconomic deprivation, in a free at point-of-access Scottish National Healthcare Service. Our findings indicate a previously unreported association with socioeconomic deprivation and ocular trauma necessitating admission to hospital in a population-based study within a national healthcare system that provides a free universal health service. Health inequalities within a free-access healthcare system have been well documented in other forms of trauma, such as head trauma,^19^ ^20^ hand injuries,^21^ maxillofacial injuries^22^ and all trauma.^23^

There was no evidence of a significant association between differences in drive or public transport time to healthcare services and the time to presentation of injury, suggesting that there is no evidence that patients are delaying their admissions due to poor geographical access. Other barriers to healthcare utilisation such as healthcare-seeking behaviour and motivation, logistical and mobility difficulties, lack of knowledge, stigma and patient anxiety may be contributory.

Our study also showed that there was a higher frequency of injuries in patients who were intoxicated at time of injury and in those from more deprived areas. Similar trends of socioeconomic inequalities in alcohol-related mortality have been widely reported.^24–26^ In the UK, alcohol-related mortality is five times more frequent among men living in the most deprived neighbourhoods compared with those in the least deprived areas.^27^ The link between socioeconomic deprivation and substance/alcohol misuse could be partly explained by ‘social selection or drift’,^28^ which relates to lower earning potential or employability in individuals with alcohol/drug misuse, and therefore higher likelihood of living in deprived areas or homelessness, and thereby ‘drifting down the social hierarchy’.^29^

The strengths of our study are the prospective design of the study and the ability to capture geographical data on accessibility of health services. Additionally, we were able to enhance ascertainment of eligible cases through the well-established BOSU that has facilitated numerous published UK-based national ophthalmic studies.^30^ ^31^ Limitations of our study include the possibility of under-reporting of cases^2^ ^8^ which is inherent in routine surveillance schemes using a single source of case identification. In addition, patients may not necessarily present to their GP as the first point of contact to healthcare services, and patients in socioeconomically deprived backgrounds are not as likely to be registered to their nearest GP. There are also limitations to ascertaining the exposure to intoxication, whether it is drug- or alcohol-related intoxication, and potential recall bias. Overall, given the small numbers of a total cohort of 104 patients, and particularly with a subgroup of only 25 patients who were reported to be ‘intoxicated’, we are cautious to report that our findings are suggestive, but not conclusive of an ‘intoxication effect’; therefore, larger prospective studies with objective parameters would be warranted.

Our study has provided a comprehensive insight into socioeconomic inequalities of ocular trauma in Scotland. This study identifies the communities most at risk of ocular trauma who would benefit from implementation of strategic prevention and intervention strategies such as additional funding towards educational programmes in deprived areas to highlight the awareness of risk contributing to ocular trauma. Eye health and ocular trauma should not be overlooked in broader strategies to address health inequalities.
